# DSCAM differentially modulates pre- and postsynaptic structural and functional central connectivity during visual system wiring

**DOI:** 10.1186/s13064-018-0118-5

**Published:** 2018-09-15

**Authors:** Rommel A. Santos, Ariel J. C. Fuertes, Ginger Short, Kevin C. Donohue, Hanjuan Shao, Julian Quintanilla, Parinaz Malakzadeh, Susana Cohen-Cory

**Affiliations:** Department of Neurobiology and Behavior, University of California, Irvine, 2205 McGaugh Hall, Irvine, CA 92697-4550 USA

**Keywords:** DSCAM, In vivo imaging, Dendritogenesis, Axon branching, Optic tectum, Retina, Retinal ganglion cell, Bipolar cell, *Xenopus laevis*

## Abstract

**Background:**

Proper patterning of dendritic and axonal arbors is a critical step in the formation of functional neuronal circuits. Developing circuits rely on an array of molecular cues to shape arbor morphology, but the underlying mechanisms guiding the structural formation and interconnectivity of pre- and postsynaptic arbors in real time remain unclear. Here we explore how Down syndrome cell adhesion molecule (DSCAM) differentially shapes the dendritic morphology of central neurons and their presynaptic retinal ganglion cell (RGC) axons in the developing vertebrate visual system.

**Methods:**

The cell-autonomous role of DSCAM, in tectal neurons and in RGCs, was examined using targeted single-cell knockdown and overexpression approaches in developing *Xenopus laevis* tadpoles. Axonal arbors of RGCs and dendritic arbors of tectal neurons were visualized using real-time in vivo confocal microscopy imaging over the course of 3 days.

**Results:**

In the *Xenopus* visual system, DSCAM immunoreactivity is present in RGCs, cells in the optic tectum and the tectal neuropil at the time retinotectal synaptic connections are made. Downregulating DSCAM in tectal neurons significantly increased dendritic growth and branching rates while inducing dendrites to take on tortuous paths. Overexpression of DSCAM, in contrast, reduced dendritic branching and growth rate. Functional deficits mediated by tectal DSCAM knockdown were examined using visually guided behavioral assays in swimming tadpoles, revealing irregular behavioral responses to visual stimulus. Functional deficits in visual behavior also corresponded with changes in VGLUT/VGAT expression, markers of excitatory and inhibitory transmission, in the tectum. Conversely, single-cell DSCAM knockdown in the retina revealed that RGC axon arborization at the target is influenced by DSCAM, where axons grew at a slower rate and remained relatively simple. In the retina, dendritic arbors of RGCs were not affected by the reduction of DSCAM expression.

**Conclusions:**

Together, our observations implicate DSCAM in the control of both pre- and postsynaptic structural and functional connectivity in the developing retinotectal circuit, where it primarily acts as a neuronal brake to limit and guide postsynaptic dendrite growth of tectal neurons while it also facilitates arborization of presynaptic RGC axons cell autonomously.

## Background

Wiring functional neuronal circuits during embryonic development involves a coordinated effort to spatially organize dendritic and axonal arbors into one cohesive circuit. The spatial pattern of dendritic arbors is critical to the neuron’s input, so that incoming information from afferent axons is efficiently integrated [1]. Neuronal arbors can adopt an array of patterns to suit their connectivity. For a notable example, individual branches in a dendritic arbor avoid aggregating with neighboring sister branches stemming from the same neuron, a phenotype referred to as self-avoidance. Axon arbors also exhibit self-avoidance [2]. Extensive studies from the last decade have shown that Down Syndrome Cell Adhesion Molecules (DSCAMs) play a multifaceted role in shaping circuit connections. DSCAMs are key players mediating not only in self-avoidant dendritic patterning, but also neuronal arbor tiling, axon guidance, and neuronal fasciculation [1, 3–6].

In *Drosophila*, DSCAM acts as a contact-dependent adhesion molecule with over 38,000 alternatively spliced isoforms coordinating the self-avoidant patterning of neuronal dendritic and axonal arbors [5–8]. While genetic conservation appears to exist between vertebrate DSCAM and *Drosophila* DSCAMs, emerging roles for vertebrate DSCAM are beginning to be uncovered. In DSCAM knockout mice, retinal ganglion cells (RGCs) have severe defects in dendritic self-avoidance phenotypes [9–11]. Studies in the chick retina have shown that DSCAM plays a role in synapse formation by promoting the targeting of RGC dendrites and bipolar cell axons to the same layer [12]. Additionally, recent evidence has demonstrated that DSCAM actively regulates circuit level plasticity by inhibiting dendritic arbor growth and receptive field size of mature retinal bipolar cells [4]. These findings suggest that DSCAM has a prominent role in wiring and maintaining the intricate arbor connections of retinal circuits in the eye. Its role, however, in orchestrating the interconnectivity between pre- and post-synaptic arbors of circuits in the brain, particularly at higher visual centers, remains largely unknown. For this reason, we aimed to test the hypothesis that DSCAM directs retinotectal synaptic connectivity by guiding the structural arborization and development of pre- and postsynaptic arbors. Additionally, we addressed whether DSCAM gives rise to proper functional visual circuits.

To understand the cell-autonomous actions of DSCAM in the retinotectal circuit, we used targeted single-cell knockdown and overexpression approaches to alter DSCAM expression levels in *Xenopus laevis* tadpoles. Structural changes in the neuronal arbor in response to alterations in DSCAM levels were observed by in vivo confocal microscopy imaging. Our findings reveal that decreasing levels of DSCAM in tectal neurons surprisingly does not affect dendritic self-avoidant patterning. Instead, individual dendrites of neurons with DSCAM knockdown took on a tortuous meandering pathway. Additionally, tectal neurons exhibited exuberant dendritic arbor growth within 24 h of DSCAM knockdown, an effect that became more robust over a three-day period of imaging. Overexpression of *Xenopus* DSCAM in single tectal neurons, in contrast, resulted in stunted dendrite arbor development. Tectal neurons overexpressing DSCAM had a significantly shorter total dendrite arbor length and fewer branches compared to controls. In contrast to tectal neurons, axons of RGCs with DSCAM knockdown branched at a slower rate over the course of 3 days when compared to control axons but retained their self-avoidant phenotypes while dendritic arbor morphology of developing RGCs was unaffected by altered DSCAM expression. Together these observations indicate that DSCAM can shape retinotectal connectivity by acting cell autonomously in multiple ways; by limiting dendritic differentiation of postsynaptic central neurons while independently facilitating retinal axon arbor growth at the postsynaptic target. Our observations that DSCAM tectal knockdown elicits deficits in the tadpole’s ability to process visual information further indicate that structural changes mediated by DSCAM can also influence functional connectivity in the developing vertebrate nervous system.

## Methods

### Animals

*Xenopus laevis* tadpoles were obtained by in vitro fertilization of oocytes from adult females primed with human chorionic gonadotropin and raised in rearing solution [60 mM NaCl, 0.67 mM KCl, 0.34 mM Ca(NO_3_)_2_, 0.83 mM MgSO_4_, 10 mM HEPES, pH 7.4, and 40 mg/l gentamycin] plus 0.001% phenylthiocarbamide to prevent melanocyte pigmentation. Tadpoles were anesthetized during experimental manipulations with 0.05% tricaine methanesulfonate (Finquel; Argent Laboratories, Redmond, WA, USA). Staging was performed according to Nieuwkoop and Faber [13]. Animal procedures were approved by the Institutional Animal Care and Use Committee of the University of California, Irvine (Animal Welfare Assurance Number A3416–01).

### Immunohistochemistry and western blot analysis

Stage 45 tadpoles were euthanized with tricaine methanesulfonate and fixed in 4% paraformaldehyde in PB, pH 7.5, for 2 h. For coronal sections, tadpoles were cryoprotected in 30% sucrose overnight and embedded in OCT compound (Sakura Finetek, Torrance, CA, USA), and 40-μm cryostat sections were obtained. Coronal sections at the level of the optic tectum were incubated with a rabbit polyclonal antibody against the middle region of human DSCAM (1:1000 dilution; Aviva System, San Diego, CA, USA). DSCAM primary antibodies were visualized using goat anti-rabbit Alexa 488 secondary antibodies (1:500 dilution; Invitrogen, Eugene, OR, USA). The specificity of DSCAM antibodies (1:500 dilution) to recognize endogenous *Xenopus* DSCAM was further tested and confirmed by Western blot analysis: a band of ∼ 220 kDa was detected by anti-DSCAM antibodies in stage 38, 41, 47 *Xenopus* brain lysates.

Immunohistochemistry was also used to confirm downregulation of DSCAM expression by lissamine-tagged morpholino anti-sense oligonucleotide (MO) treatment (300 nmol, Genetools, Philomath, OR, USA). Morpholino-injected embryos were raised until stage 38 or 42 (3 to 4 days-post fertilization) to be fixed and analyzed by immunohistochemistry for DSCAM as above. To obtain a relative change in DSCAM immunoreactivity, fluorescence intensity of Alexa 488 immunoreactivity was measured from at least five regions of interest (ROI = 30 × 30 μm) per brain hemisphere, or retina, where fluorescein-tagged DSCAM was localized and compared to the corresponding ROIs in the contralateral brain hemisphere, or adjacent retinal area, without MO label.

Immunohistochemistry of stage 45 tadpoles injected with fluorescein-tagged DSCAM or Control MO at the four-cell stage or electroporated at stage 43 was also used to determine synaptic changes by immunostaining with antibodies to vesicular glutamate transporter 2 (VGLUT2; 1:200 dilution, guinea pig polyclonal antibody; EMD Millipore, #AB2251) and vesicular GABA transporter (VGAT; 1:100 dilution, rabbit polyclonal antibody; Phosphosolutions, #2100-VGAT). Alexa 568 anti-rabbit and Alexa 633 anti-chick secondary antibodies were used to visualize VGLUT and VGAT immunoreactivity respectively. To obtain a change in VGLUT or VGAT ratio, fluorescence intensity was quantified in individual cryostat sections imaged by confocal microscopy at the three wavelengths from at least five regions of interest (each ROI = 30 × 30 μm) per brain hemisphere where fluorescein-tagged MO was localized. Fluorescence intensity values for each wavelength were normalized for each brain section to compare fluorescence intensity in the area/hemisphere without MO label (contralateral side) with the corresponding area/hemisphere with the MO label (ipsilateral side). Specifically, to standardize fluorescence intensity across sections and animals, fluorescence intensity measures were normalized per brain section by averaging the pixel intensity values for all ROIs in the hemisphere without MO label (contralateral side) in that section, normalizing the average intensity value of the “contralateral side” to 100, and recalculating pixel intensity values for each individual ROI (contralateral and ipsilateral sides) within each brain section. Normalized values for six individual sections, each from an individual tadpole per treatment, obtained from two independent experiments were used for statistical comparison (Student t-test).

### Transfection of Morpholinos or plasmids

Downregulation of DSCAM expression was performed using lissamine-tagged morpholino anti-sense oligonucleotides (300 nmol, Genetools, Philomath, OR, USA) to block protein translation. A morpholino (MO) against *Xenopus laevis Dscam* mRNA was designed with the sequence 5′-ACATATAAGACTTCGACAGAGACGT-3′. 10-nL volume of DSCAM MO was injected into the two light-shaded blastopores of a 4-cell stage embryo using a pressurized microinjector (Picospritzer, General Valve). A standard lissamine-tagged control morpholino oligonucleotide with the following sequence 5′-CCTCTTACCTCAgTTACAATTTATA-3′ was used for control comparisons. Morpholino-injected embryos were raised until stage 38 or 42 (3 to 4 days-post fertilization) to be fixed and analyzed by immunohistochemistry for DSCAM as above. Targeted downregulation of DSCAM expression in developing tectal neurons or in RGCs was achieved using single-cell electroporation in developing *Xenopus* tadpoles [14]. Prior to electroporation, tadpoles were anesthetized with 0.05% tricaine methanesulfonate. A CUY-21 edit stimulator was used to electroporate and transfect individual tectal neurons or RGCs of stage 43 tadpoles (20 V, 1 ms pulse duration on, 1 ms pulse duration off, set to repeat 99 times). Tectal neurons or RGCs were electroporated with lissamine-tagged DSCAM MO (150 nM pipette concentration) and a cell-filling dye Alexa Fluor 488 Dextran, 3000 MW (2 mg/111 μl pipette concentration, Invitrogen, Eugene, OR, USA). Reagents were loaded onto an aluminosilicate electrode (AF100–64-10, 1.00 mm, 0.64 mm, 10 cm) with a pulled tapered-tip with an opening of about 0.5 μm. Neurons transfected with a standard lissamine-tagged control MO (150 nM pipette concentration) and 488 dextran were used as a control comparison with DSCAM MO transfected neurons. Co-transfections of lissamine-tagged morpholinos and Alexa 488 dextran was confirmed via fluorescence microscopy. For DSCAM downregulation in retina, Control or DSCAM MO was pressure injected into both the left and right eyes of anesthetized stage 42 tadpoles. Directly after the microinjection, tadpoles were electroporated with 20 V at both normal polarity and reversed polarity with the CUY-21 edit stimulator. Tadpoles were then left in a 12-h light-dark cycle at 22 °C until stage 45 (~ 2 days later).

**Fig. 1:**
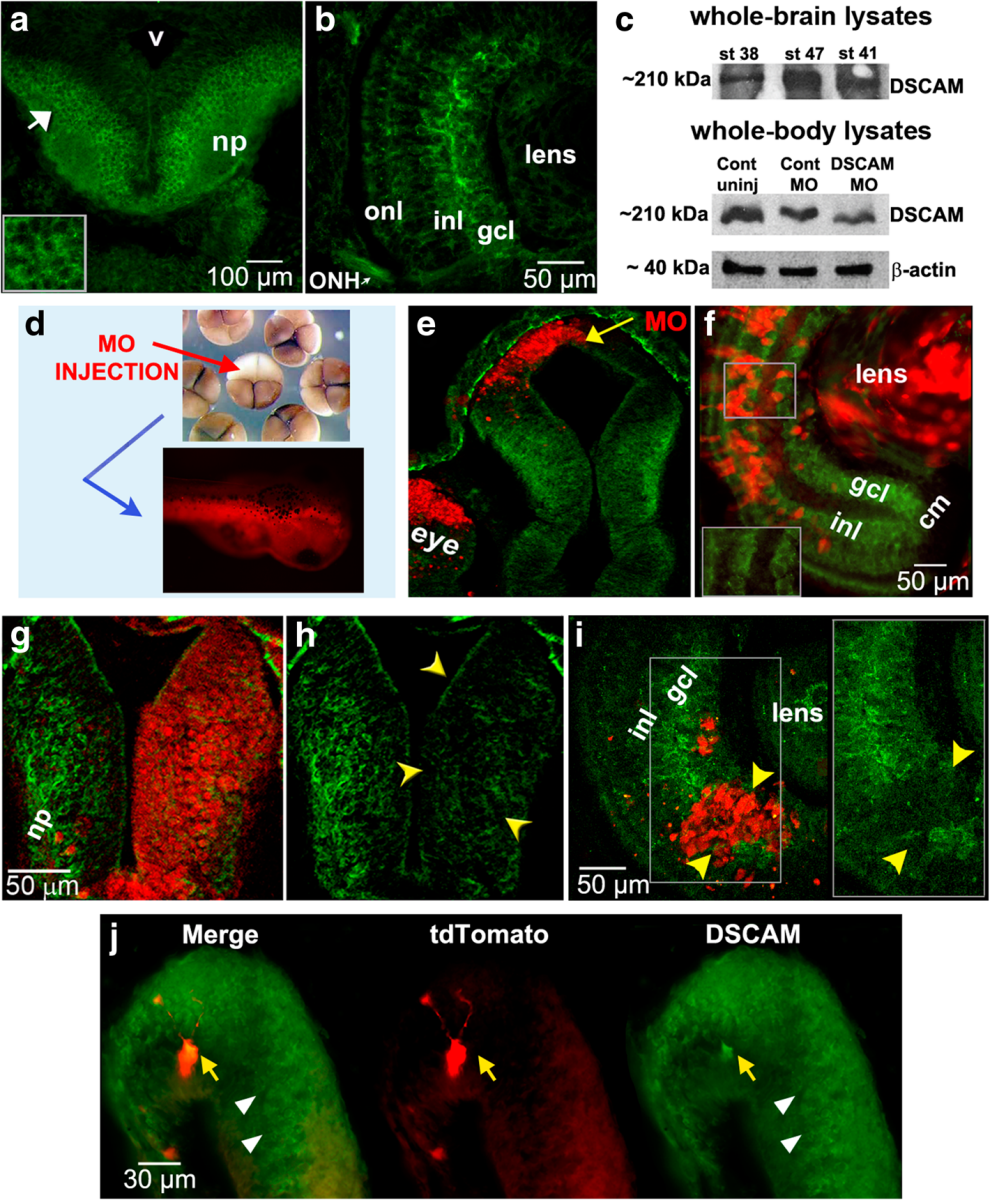
DSCAM expression in the developing *Xenopus* visual system and morpholino oligonucleotide-mediated knockdown. Immunostaining reveals patterns of DSCAM expression in the retina and tectum of developing *Xenopus* tadpoles. (**a**, **b**) DSCAM immunoreactivity (*green*) localizes to the midbrain (**a**) and retina (**b***)* of stage 40 tadpoles. In the midbrain optic tectum DSCAM immunoreactivity is localized to postmitotic cell bodies (white arrow and insert in **a**) and neuropil (np). In the developing retina (**b**), DSCAM immunoreactivity localizes to the inner nuclear layer (inl), ganglion cell layer (gcl) and optic nerve head (ONH). **c** Western blot analysis of whole brain lysates confirms DSCAM expression in stage 38, 41, and 47 tadpoles. Whole-embryo lysates at stage 30 show a 40% decrease in DSCAM expression after microinjection of DSCAM MO at the 2-cell stage. **d** Microinjection of lissamine-tagged DSCAM or Control MO into a light-shaded blastomere of 4-cell or 8-cell stage embryos localized the MO to cells in the eye and brain of developing tadpoles unilaterally. **e**, **f** Lissamine-tagged Control MO (*red*) did not alter DSCAM expression (*green*) in stage 38 tectum (**e**) or stage 45 retina (**f*;*** see magnified insert) by injection at the 8-cell stage. **g-i** Decreased DSCAM expression (*green*) is observed in the tectal hemisphere of stage 45 tadpole (**g***,* ***h****; yellow arrowheads*) and portion of retina of stage 40 tadpole *(****i****; see magnified insert*; *yellow arrowheads*) with DSCAM MO lissamine tag (*red*). **j** DSCAM immunostaining of stage 45 tadpole brain lipofected with plasmids coding for *Xenopus Dscam* and tdTomato. Note the increased levels of DSCAM immunoreactivity in tdTomato-labeled neuron (*yellow arrow*). The white arrowheads denote endogenous DSCAM expression. *np,* neuropil; *v*, ventricle; *MO,* morpholino; *inl,* inner nuclear layer; *gcl,* ganglion cell layer; *onl*, outer nuclear layer; *ONH*, optic nerve head, *cm,* ciliary margin. Scale bars: 100 μm in (**a**); 50 μm in (**f**, **g**, **i**); 30 μm in (**j**)

Overexpression of DSCAM in individual tectal neurons was conducted by co-electroporating pCALNL-TurboRFP and pCALNL-GFP-*Dscam* (both at 5 μg/µl pipette concentration) with pCAG-Cre:GFP (2 ng/µl pipette concentration) into the optic tectum of stage 43 embryos to sparsely label individual tectal neurons. The pCALNL-GFP-*Dscam* was constructed by amplifying the *Xenopus laevis Dscam* sequence from a pCMV-SPORT6-*Dscam* (pDONR223 vector, Source BioScience), with the following primers: forward Kpn-*Dscam* primer: 5’-CCGAGGTACCATGTTATATGACCTGCAGGA-3′, Reverse AgeI-DSCAM primer: The *Dscam* sequence was then ligated downstream of the GFP sequence of the pCALNL-GFP (Addgene plasmid # 13770), a gift from Connie Cepko [15]. The pCAG-Cre:GFP was also a gift from the Cepko lab (Addgene plasmid # 13776). The pCALNL-TurboRFP plasmid was generously provided by Yoshiaki Tagawa [16]. Co-transfections of sparsely labeled neurons with a pCS2-eGFP and the pCMV-SPORT6-*Dscam* plasmid were also performed by lipofecting the brain primordia of stage 22 tadpoles as before [17, 18]. Anesthetized tadpoles were imaged at stage 45 by laser-scanning confocal microscopy. Overexpression of DSCAM was further confirmed by immunohistochemistry after imaging (see Fig. 1j).

### In vivo confocal microscopy imaging

Stage 45 tadpoles were anesthetized with 0.05% tricaine methanesulfonate prior to imaging, were mounted in a custom-made sylgard chamber during imaging, and were then allowed to recover in fresh rearing solution immediately after imaging. Neurons co-transfected with lissamine-tagged morpholinos and Alexa 488 dextran were imaged in real time using an LSM780 confocal microscope (Zeiss) over the course of 3 days, at 24-h intervals. The LSM 780 confocal microscope is equipped with a MaiTai Ti:Sapphire multiphoton laser system. A two-photon wavelength of 760 to 780 was used to image the Alexa 488 cell-filling dye in tectal neurons and RGC axons in the midbrain. Neurons co-transfected with pCALNL-TurboRFP and pCALNL-GFP-*Dscam* were imaged using a multiphoton LSM780 confocal microscope starting 48 h after electroporation over the course of 3 days, at 24-h time intervals. pCALNL-TurboRFP and pCALNL-GFP-*Dscam* co-transfected neurons were imaged with Argon and HeNe lasers simultaneously. For analysis of RGC and bipolar cell dendritic morphologies, tadpoles with retinal MO transfections were reared until stage 45 (48 h post-injection), euthanized with tricaine methanesulfonate, then fixed in 4% paraformaldehyde overnight at 4 °C and transferred to 30% sucrose for at least 1 h to overnight in 4 °C. Tadpoles were immersed in OCT embedding compound and 60 μm thick cryostat sections were obtained. Slides were then coverslipped with ProLong™ Gold Antifade Mountant with DAPI to label nuclei and differentiate between the retinal layers. For arbor analysis, images of the retina were taken with a 63× oil-immersion objective using a Zeiss Pascal laser scanning confocal microscope equipped with a HeNe laser. Images were collected in a 0.5 μm interval throughout the extent of the dendritic arbor (z-axis).

### Neuronal arbor analysis

In brief, three-dimensional images of fluorescently-labeled dendritic arbors were manually reconstructed using a Neuromantic tracing software blind to treatment. Alexa 488 dextran-labeled RGC axon arbors were also reconstructed using Neuromantic. Each dendritic or axonal arbor was reconstructed plane-by-plane from the image stack and was then analyzed using the Neuromantic software. Branch tips were identified as the terminal ends of primary dendrites or axons. Primary branches were identified as projections stemming from the soma. The total arbor lengths, branches, and branch tips of the cells were thresholded, binarized, and skeletonized with the Neuromantic software so that the soma perimeter and dendrites were represented as a single pixel width. Processes of more than 5 μm in length were considered branches, while processes less than 5 μm were categorized as filopodia. Statistical analysis was performed as described [19]. Additionally, ImageJ was used for three-dimensional Sholl analysis of reconstructed arbors to quantify the number of proximal and distal branches from a given neuron. A radius step size of 10 μm intervals were used for both dendritic and axonal arbor measurements. For tectal neuron dendritic arbors, the number of intersections was quantified starting at the main branch point stemming from the soma. For axonal arbors, Sholl analysis was quantified 5 μm from the main branch point of the primary axonal stem. Sholl branch-tip distributions were compared across experimental groups and two-way ANOVA statistical analysis of data was performed. Neuromantic data and Sholl analysis results were considered significant in comparison to control as follows: *p ≤ 0.05, **p ≤ 0.005, ***p ≤ 0.001, unless otherwise indicated on the graph with bars marking additional significant comparisons.

### Visual avoidance task

Stage 45 tadpoles were placed in a 60 mm × 20 mm clear plastic petri dish, with darkened walls, filled to a depth of 1 cm with modified rearing solution at room temperature. The dish was placed on a CRT monitor screen and a solid, opaque box was placed over the monitor to eliminate outside light. A camera was affixed to the opening at the top of the box for video recording. Visual stimuli were produced by a custom-written Matlab program (MathWorks, Natick, MA, USA) generously donated by Dr. Carlos Aizenman, Brown University. A black circle with radius 0.3 mm was projected in the center of a circle on a white background. This size was found to produce optimal responses to the stimulus as shown in [19]. The circle was then manually directed to collide with the path of the swimming tadpole every 30 s for six trials. The tadpole’s responses to the circle, when the dot approached the tadpole and when the dot returned to the dish center, were analyzed blind to treatment with frame-by-frame replay of recorded responses. Tadpoles were observed to both freeze and swim away by altering their direction, speed, or both when presented with stimuli. These responses were counted as visual reactions to the stimuli. Failure to move away from the circle or a lack of freezing behavior prior to when the circle encountered the tadpole was considered a failure to respond. Experiments were performed during the 12-h light cycle. Treatments were identical to those of in vivo imaging studies with the exception that tadpoles were injected in the ventricle and laterally in the subpial space overlying both tectal hemispheres. Only tadpoles that responded to at least 50% of the visual stimuli at 0 h were included in the analysis. The behavior of a total of 16–26 tadpoles was analyzed per condition: 16 controls uninjected, 25 vehicle-injected, 14 Control MO-treated, 26 DSCAM MO-treated. Student’s t-tests and one-way ANOVA with Tukey’s multiple comparison tests were used for the statistical analysis of the data. Results of behavioral analysis were considered significant as follows: *p ≤ 0.05, **p ≤ 0.005, ***p ≤ 0.001.

## Results

### Patterns of DSCAM expression in the *Xenopus* retina and optic tectum during visual circuit development

Immunohistochemistry of coronal brain sections reveal that DSCAM is expressed both in the retina and optic tectum of *Xenopus* tadpoles at the time that RGCs differentiate and project their axons out of the eye and into the brain (stages 38–40; Fig. 1a, b, e). Western blot analysis of whole-brain lysates also confirmed expression of DSCAM in stage 38 to stage 47 tadpoles (Fig. 1c). Expression in the retina and optic tectum also occurs during the time when retinotectal synaptic connections begin to be made (stage 45; Fig. 1f, g). In the midbrain optic tectum DSCAM is expressed in the cell body layer where mature neurons localize as well as in the neuropil, where dendrites and axons establish functional synaptic connections (Fig. 1a, g). Expression of DSCAM in the ganglion cell layer (gcl), inner plexiform layer (ipl) and inner nuclear layer (inl) in the *Xenopus* retina (Fig. 1b, f, i) is consistent with expression patterns and roles for DSCAM in other vertebrate species [4, 11, 12, 20].

To examine the impact of downregulating DSCAM levels during *Xenopus* visual circuit development, we utilized a morpholino (MO) anti-sense oligonucleotide targeted against endogenous *Xenopus laevis Dscam* mRNA to interfere with protein translation. To test for the specificity of the MO, we injected control or *Xenopus*-specific DSCAM MO into a single blastomere of 2-cell or 4-cell stage embryos and visualized changes in expression by western blot and by immunostaining tadpoles with antibodies to DSCAM at different developmental stages (Fig. 1c-i). *Xenopus* DSCAM morphants developed normally and were as healthy as controls. MO microinjections into a light-shaded blastomere of a 4-cell stage embryo (or two light-shaded blastomere of 8-cell stage embryos) restricted the MO to only one side of the organism’s body and resulted in tadpoles with MO localized to the eye and midbrain of stage 38 tadpoles (Unilaterally, Fig. 1d). DSCAM morphants showed significant changes in brain and retinal DSCAM expression (Fig. 1c, g, h, i). Tadpoles with DSCAM MO label (lissamine-tagged MO) localized to the neuropil, where retinotectal synaptic connections are formed, showed a 59.16% average fluorescence intensity reduction in DSCAM immunoreactivity (Fig. 1g, h). Similarly, DSCAM MO presence in the RGC layer of the retina correlated with a 59.6% reduction in DSCAM antibody fluorescence intensity (Fig. 1i, see insert). In contrast, injection of Control MO resulted in an 8.9% average fluorescence intensity reduction of DSCAM immunoreactivity in the RGC layer (Fig. 1f, see insert) and a 0% reduction within the tectal neuropil (data not shown). Consistent with these findings, western blot analysis of DSCAM morphant stage 30 tadpoles revealed a 40% decrease in DSCAM protein levels (Fig. 1c). These observations confirm our MO loss-of-function approach and indicate that DSCAM knockdown is specific and affects only DSCAM morphant neurons.

### Developing tectal neurons exhibit exuberant dendrite growth and extend more proximal and distal branches in response to DSCAM downregulation

**Fig. 2:**
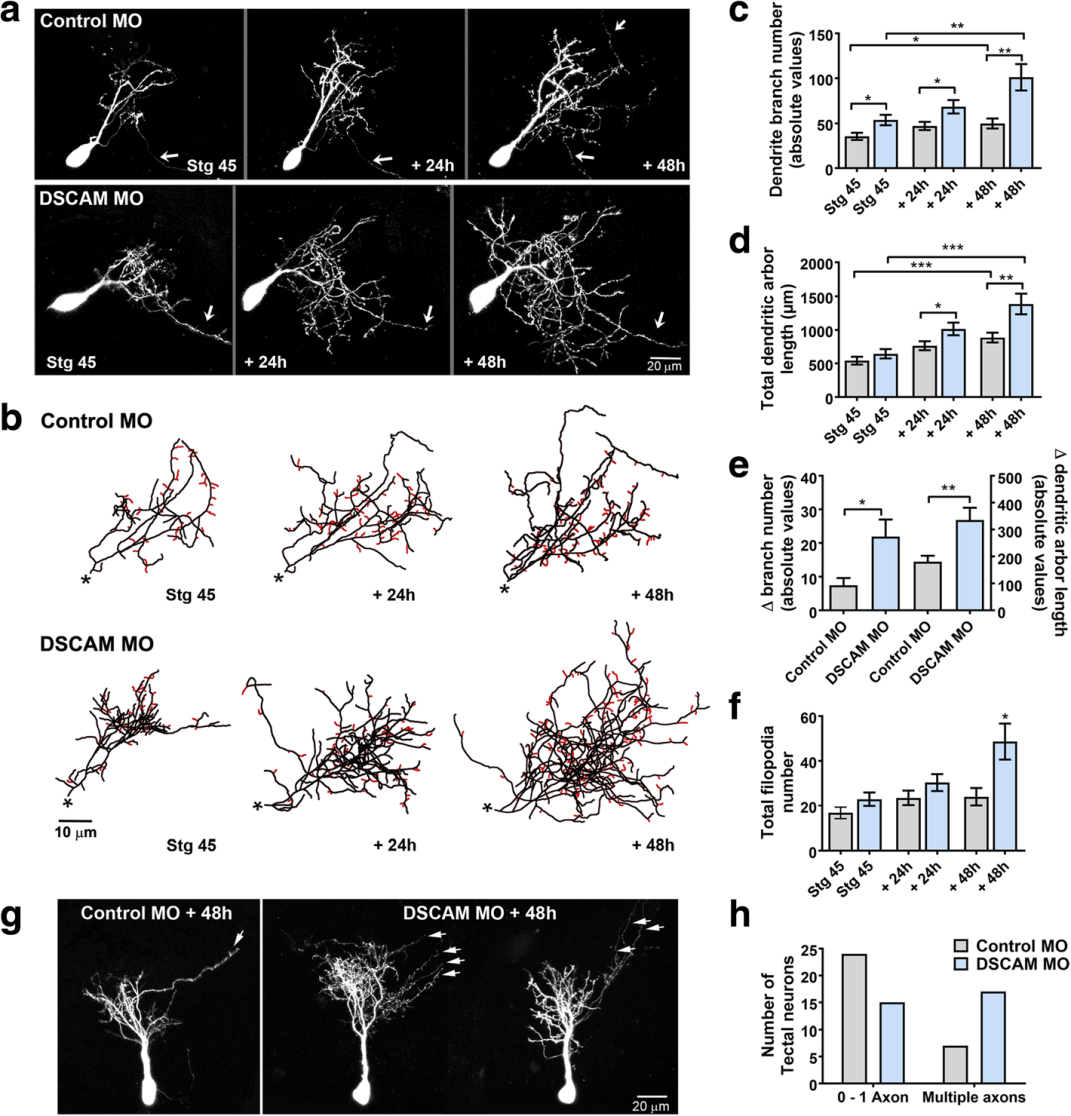
Single-cell DSCAM knockdown increases the branching and growth of tectal neurons in vivo. **a** Sample neurons of stage 45 tadpoles transfected with Alexa 488 dextran and lissamine-tagged Control MO or DSCAM MO and imaged in vivo by two-photon confocal microscopy over the course of 3 days. **b** Dendritic arbors were digitally reconstructed in three-dimensions using the Neuromantic tracing software. Filopodia, processes of less than 5 μm were manually measured and highlighted in red. **c** Dendritic arbors of neurons with DSCAM MO had significantly a higher number of branches than controls at each imaging time point, (**d**) and a higher total arbor length at 28-h and 48-h after initial imaging compared to controls. **e** Quantifying the rate of branch addition and the increase in total dendritic arbor length reveals that tectal neurons with DSCAM MO grow at a more robust and faster rate than controls (Student’s-t-test). **b**, **f** Tectal neurons had significantly more filopodia compared to controls by 48 h after initial imaging only. **g**, **h** Tectal neurons with DSCAM MO also extended significantly more axons (marked by the white arrows) than controls. Control MO (*n* = 31), DSCAM MO (*n* = 31). In **c***-***e**, comparisons are by Two-way ANOVA and Student’s t-test. * *p* ≤ 0.05, ** *p* ≤ 0.005, *** *p* ≤ 0.001. In **h**, statistical comparison was by Fisher’s Exact Test, *p* = 0.0192. Scale bars: 20 μm in (**a** & **g**); 10 μm in (**b**)

To define direct cellular actions of DSCAM on tectal neurons, single-cell electroporation of lissamine-tagged MOs together with Alexa 488 dextran in stage 43 tadpoles was used to acutely downregulate DSCAM expression cell-autonomously. Individual tectal neurons were imaged in vivo using two-photon confocal microscopy to visualize neuronal morphology 24 h after MO transfection (stage 45 tadpoles). Tadpoles were imaged again 24 and 48 h after initial imaging. Single-cell DSCAM MO electroporation resulted in tectal neurons with exuberant dendritic arbor growth, an effect that was sustained over the entire imaging period (Fig. 2a-c). Three-dimensional reconstruction and quantitative analysis revealed that neurons transfected with DSCAM MO had significantly higher dendrite branch number at each imaging time point (Fig. 2c) and higher total dendrite arbor length by 48 h after initial imaging when compared to controls (Fig. 2d). Neurons with DSCAM MO-mediated knockdown also grew at a faster rate than controls (Fig. 2e). To further differentiate whether DSCAM downregulation increases branch and/or filopodia number, processes less than 5 μm were counted from each individual neuron at every imaging time point (filopodia marked red; Fig. 2b). This analysis revealed that tectal neurons with DSCAM knockdown possessed significantly more filopodia by 48 h after initial imaging (Fig. 2b, f), while the total number of branches was significantly increased at all imaging time points when compared to controls (stage 45: Control MO 19.53 ± 1.87, DSCAM MO 27.06 ± 2.63, *p* = 0.024; + 24 h: Control MO 23.78 ± 1.9, DSCAM MO 36.0 ± 3.78, *p* = 0.0059; + 48 h: Control MO 26.86 ± 2.5, DSCAM MO 48.5 ± 6.3, *p* = 0.003). These results indicate that the increase in total branching we observed from DSCAM downregulation is mostly a result from an increase in dendritic branch number and, to a smaller extent, an increase in filopodia number. In addition to the effect of DSCAM knockdown on dendrite number and length, we observed that the proportion of neurons that extended more than one axon was increased after DSCAM MO-mediated knockdown when compared with controls (Fig. 2g, h).

**Fig. 3:**
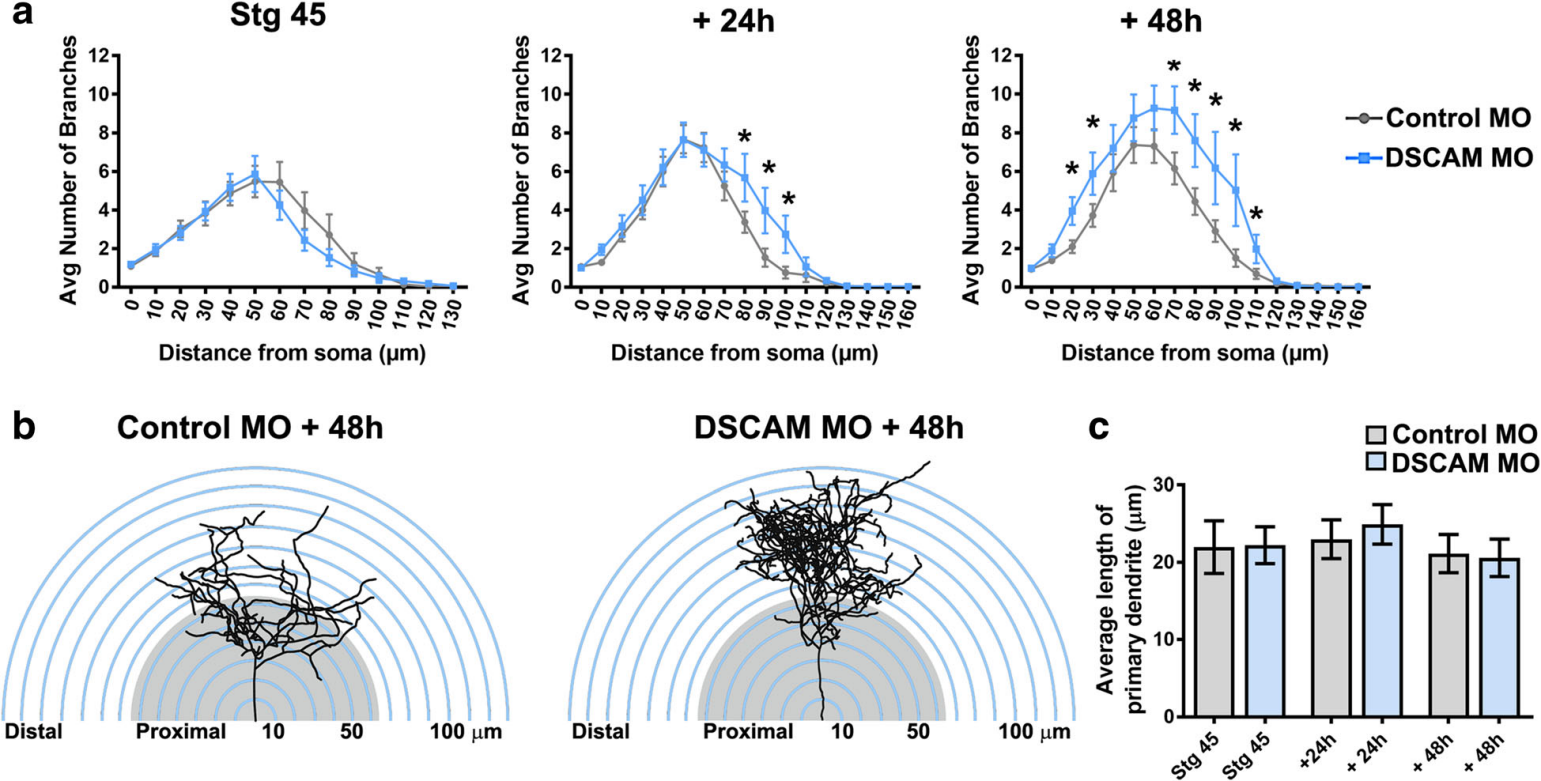
Exuberant dendrite arbor growth after DSCAM knockout. **a** Three-dimensional Sholl analysis of proximal and distal dendrites of tectal neurons transfected with either DSCAM MO or Control MO was used as a measure of dendritic arbor complexity. The number of proximal and distal branch intersections was measured for neurons in stage 45 tadpoles and 24 h and 48 h after initial imaging. **b** Tracings of representative neurons showing proximal vs distal branch distribution within a spherical Sholl-ring. **c** The length of the primary dendrite of neurons with DSCAM downregulation was similar to that of controls at each imaging time point. Control MO *n* = 31, DSCAM MO *n* = 31. Two-way ANOVA, error bars indicate mean ± SEM. *p < 0.05

Sholl analysis was used as an additional measure to understand the effects of DSCAM downregulation on dendritic arbor morphology and complexity of tectal neurons [21, 22]. Sholl analysis measured the number of dendrites, without considering filopodia, that intersected a series of spherical circles spaced at 10 μm ring intervals for each neuron analyzed in three-dimensions. Our analysis revealed that by 24 h after initial imaging, tectal neurons with DSCAM downregulation had significantly more distal branch intersections (70 to 110 μm from the soma) compared to controls (Fig. 3a, b). By 48 h after initial imaging, neurons with DSCAM MO-mediated knockdown had a significant increase in branch intersections both proximally and distally from the soma (20 to 110 μm) relative to controls. To ascertain that the increase in the proportion of distal dendrites was not a result of a primary dendrite growing longer rather than extending new branches, we measured the length of the primary dendrites of neurons treated with DSCAM MO and control MO. There were no significant differences in primary dendrite length between neurons treated with DSCAM MO compared to controls (Fig. 3c). Together, these results indicate that knockdown of DSCAM positively regulates the branching and complexity of tectal neuron dendritic arbors.

### Dendrites of neurons with DSCAM downregulation grow in highly tortuous meandering paths

**Fig. 4:**
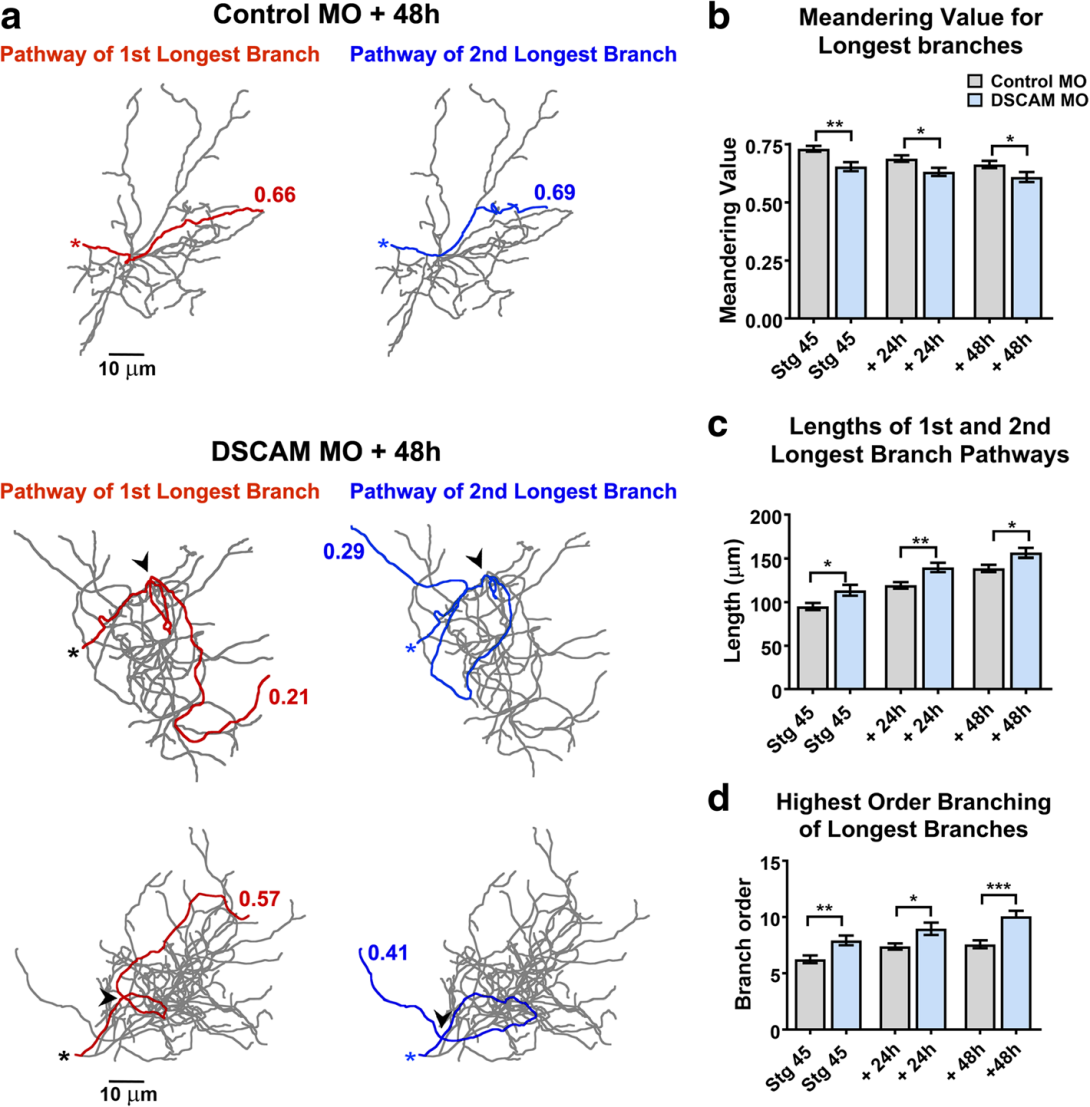
Dendrites of tectal neurons with DSCAM downregulation take tortuous meandering paths. **a** Tracings of sample neurons transfected with Control or DSCAM MO and imaged 48 h after initial imaging. For the quantification of dendritic pathway turning the 1st and 2nd longest individual branches of reconstructed neurons were measured three-dimensionally (Control MO *n* = 62 dendrites, DSCAM MO *n* = 62 dendrites) using the Neuromantic software meandering contraction value which quantifies bends and turns in a scale from 0 to 1. Here, the pathways of the two longest branches for each sample neuron are highlighted in red and blue and their corresponding contraction values are shown. Note that dendrites of neurons with DSCAM MO take abnormal turns within the dendritic arbor. **b** Individual branches of neurons with DSCAM MO showed a significantly lower contraction value at each imaging time point when compared to controls. **c** A measurement of the lengths of the 1st and 2nd longest primary branches of each neuron reveal that dendrite branches were significantly longer in neurons with DSCAM MO than in those with Control MO. **d** The longest branches in neurons with DSCAM MO also bifurcated more than controls as shown by the significant difference in their branch order number. Scale bars: 10 μm in (**a**). Statistical comparisons are by Student’s t-tests, error bars indicate mean ± SEM. *p ≤ 0.05, **p ≤ 0.005

Alterations in DSCAM expression result in errors in dendrite self-avoidance in *Drosophila* and in mature retinal neurons of DSCAM knockout mice [1, 4, 23–25]. We observed no perturbations in dendritic self-avoidance among *Xenopus* tectal neurons after MO-mediated DSCAM knockdown. Specifically, analysis of dendritic arbors in three-dimensional space using the Neuromantic software 3D viewer (where one can rotate and view tracings of reconstructed neurons at numerous angles through a 360° field of view) showed no fasciculation or crossing contact among sister dendrites of either Control MO or DSCAM MO transfected neurons (data not shown). We did notice, however, that individual branches of neurons with DSCAM downregulation took on a tortuous trajectory of growth within the dendritic arbor (Fig. 4a). Tortuous projections of arbors of neurons with DSCAM downregulation were observed in longer branches. To quantify the tortuosity of dendrites, we used the Neuromantic software contraction function to analyze the meandering of individual branches from 3D reconstructed neurons imaged in vivo. For this analysis, a dendritic branch that would take on an absolute straight path would score a value of 1, while dendrites that exhibit more “bending” or angled turns along their pathway receive lower values [26]. Dendritic pathways of the 1st and 2nd longest individual branches of reconstructed neurons were analyzed three-dimensionally and were combined to obtain an average value. Figure 4a illustrates the dendritic pathways of the 1st and 2nd longest individual branches of sample reconstructed neurons and their corresponding meandering scores. The 1st and 2nd longest individual dendrites of neurons transfected with control MO had an initial average meandering value of 0.716 at stage 45, the initial imaging period, which then slightly decreased over the course of 2 days as dendrites grew and branched (Fig. 4a, b). In contrast, the individual branches of DSCAM MO transfected neurons showed a significantly lower meandering value of about 0.6 at each imaging time point compared to controls (Fig. 4a, b). This indicates that the growth directionality of individual dendrites is affected by DSCAM downregulation.

We also quantified the total length and branch order for each neuron’s 1st and 2nd longest individual branches. The tortuous meandering paths displayed by dendrites of DSCAM MO transfected neurons could have been a result of longer branches traversing longer distances and given a better chance to take angled turns. Additionally, the altered morphology displayed by the dendritic arbors of neurons transfected with DSCAM MO could result from dendrites splitting out to higher order branch number, which would also contribute to more angled turns. This analysis revealed that the 1st and 2nd longest individual branches of neurons with DSCAM MO were significantly longer than controls (Fig. 4c). Moreover, these dendrites also extended branches that split more relative to controls (Fig. 4d). Therefore, the bending of individual longer branches and their splitting into higher order branches both contributed to the altered morphology and directionality we observed in the dendrites of neurons with DSCAM knockdown.

### Cell-autonomous overexpression of DSCAM interferes with dendrite growth and differentiation of tectal neurons

**Fig. 5:**
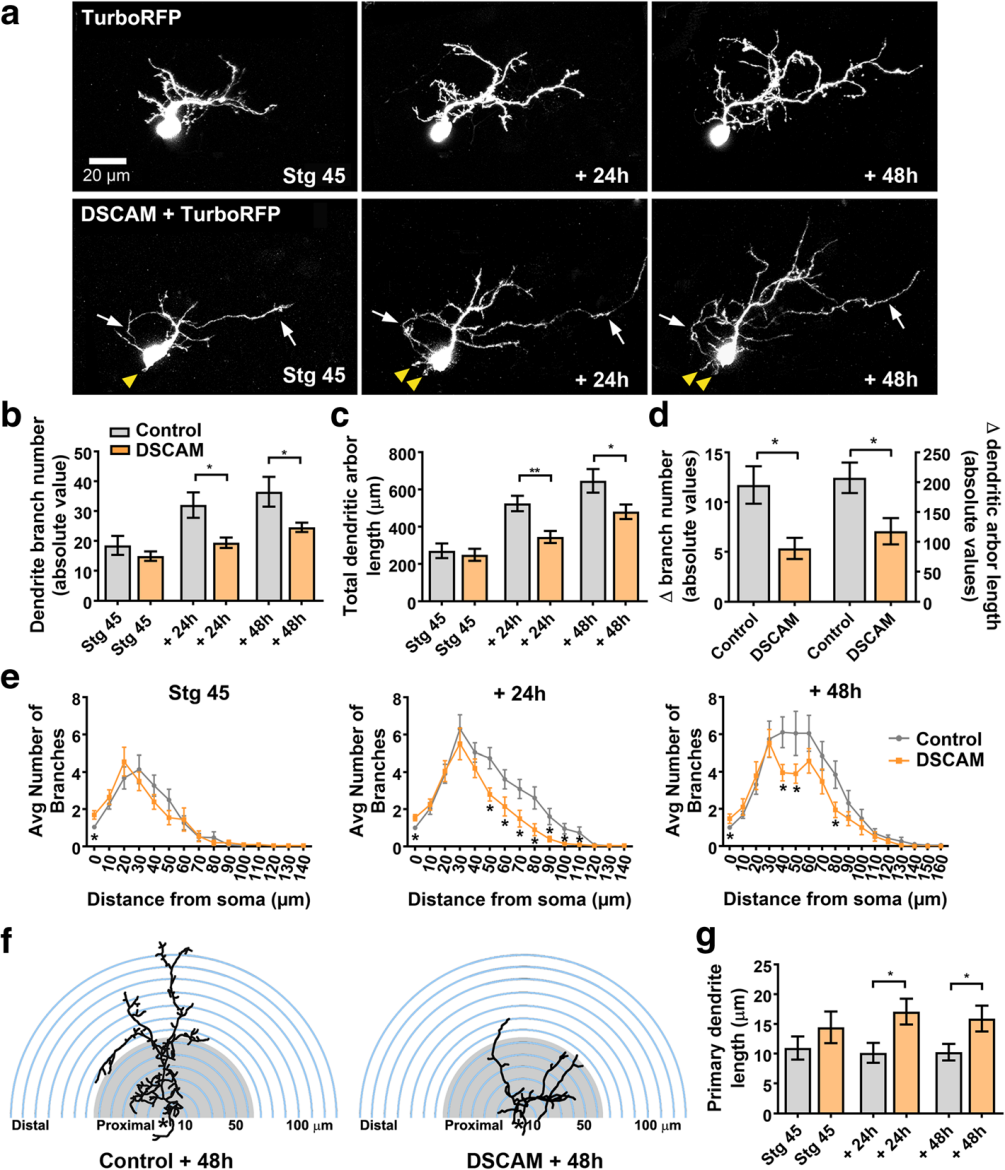
DSCAM overexpression decreases the branching and complexity of tectal neuron dendritic arbors. **a** Sample tectal neurons expressing TurboRFP or co-expressing TurboRFP and DSCAM-GFP plasmids at stage 45, and 24 and 48 h after initial imaging (*arrows* point to axons; *yellow arrowheads* point to neurites extending from soma). **b**-**d** The number of branches and total dendrite arbor length were measured for tectal neurons at stage 45, 48 h after plasmid transfection. Note that neurons overexpressing DSCAM had similar number of branches and total dendrite arbor length at the initial imaging time point but failed to increase their number of branches and their total dendrite arbor length at the rate of TurboRFP-only expressing controls. **e**, **f** Sholl analysis revealed a reduction in distal dendrite branches in neurons overexpressing DSCAM 48 h after initial imaging. **g** Note that while dendrites failed to branch, the length of the primary dendrite of neurons overexpressing DSCAM was significantly higher than controls. TurboRFP only (*n* = 22 neurons, one neuron per tadpole) or DSCAM + TurboRFP (*n* = 20 neurons, one neuron per tadpole) Comparisons are by Student’s-t-test. Error bars indicate mean ± SEM. * *p* ≤ 0.05, ** *p* ≤ 0.005, *** *p* ≤ 0.001. Scale bars: 20 μm in (**a**)

Downregulating DSCAM levels in tectal neurons triggered exuberant growth, increasing branch number and total branch length, suggesting that endogenous DSCAM is part of cellular mechanism that controls tectal neuron arbor growth in a restrictive manner. To further test this possibility, we examined cell-autonomous effects of DSCAM overexpression on tectal neuron morphology. To manipulate DSCAM expression in individual tectal neurons, we co-electroporated a Cre driver plasmid with reporter plasmids – pCALNL-TurboRFP (cell-filling dye) and a plasmid coding for *Xenopus Dscam* tagged with GFP (pCALNL-*Dscam*-GFP) in stage 42 tadpoles, a manipulation that results in sparse expression of recombinant proteins in the midbrain. Tectal neurons transfected with the Cre driver plasmid driving only pCALNL-TurboRFP were used as controls. Individual neurons were imaged by confocal microscopy 48 h after transfection, at stage 45, to allow enough time for the tectal neurons to express the chimeric and reporter proteins. Tectal neurons were further imaged 24 and 48 h after initial imaging (Fig. 5a). Quantitative analysis of three-dimensionally reconstructed dendritic arbors revealed that while tectal neurons overexpressing DSCAM had similar branch number and length at stage 45, the initial imaging period (Control 18.5 ± 3.17, *n* = 22; DSCAM-GFP 14.9 ± 1.57, *n* = 20, *p* = 0.330, Fig. 5b), they had a significantly lower dendrite branch number when compared to TurboRFP-only controls 24 h after initial imaging (Control 32.04 ± 4.26; DSCAM-GFP 19.4 ± 1.74, *p* = 0.0129, Fig. 5b), an effect that was maintained 48 h after initial imaging (Control 36.52 ± 5.007; DSCAM-GFP 24.58 ± 1.55, *p* = 0.0353, Fig. 5b). Similarly, total dendritic arbor length was significantly lower in DSCAM overexpressing neurons by 24 and 48 h after initial imaging (Fig. 5c). Quantifying the change in growth rate over the three imaging time points further demonstrates that dendritic arbors of DSCAM overexpressing neurons grew significantly slower than controls (Fig. 5d).

The effects of DSCAM overexpression on tectal neuron morphology were also examined in tadpoles co-transfected with plasmids coding for DSCAM only (rather than the chimeric construct) and GFP at stage 22, a manipulation that allowed us to alter DSCAM expression at the onset of neuronal differentiation (see Fig. 1j). Confocal microscopy imaging and analysis of neuronal morphology at stage 45 showed that tectal neurons overexpressing DSCAM had significantly fewer dendrite branches than controls (Control 20.0 ± 1.7, *n* = 11 neurons in 11 tadpoles; DSCAM 11.7 ± 1.6, *n* = 13 neurons in 13 tadpoles, *p* = 0.0024) confirming the specificity of the DSCAM overexpression effects. Together, our findings support a role for DSCAM during tectal neuron differentiation and indicate that endogenous DSCAM restricts dendritic arbor development of tectal neurons.

Sholl analysis was used as previously to assess the effects of overexpressing DSCAM on dendritic arbor morphology and complexity of tectal neurons. Overexpression of DSCAM in tectal neurons significantly reduced the number of dendritic branch intersections by 24 and 48 h after initial imaging (Fig. 5e, f). Interestingly, neurons overexpressing DSCAM had significantly more neurites extending from the soma than controls (Fig. 5a, yellow arrowheads), an effect that is reflected by the higher number of branch intersections close to the soma at stage 45 and 24 h after initial imaging (stage 45: Controls 1.045 ± 0.04545, *n* = 22, DSCAM 1.7 ± 0.2306, *n* = 20, *p* = 0.0058; at 24 h: Controls 1 ± 0, DSCAM 1.55 ± 0.1698, *p* = 0.0012). We also observed that despite having an overall simpler arbor morphology, the average length of the primary dendrite, where the dendritic tree predominantly arborizes, was significantly higher in tectal neurons overexpressing DSCAM compared to controls at 24 and 48 h after initial imaging (Fig. 5g). Together, these results support our loss-of-function experiments and indicate that endogenous DSCAM restricts the overall structural growth of higher-order dendrites of developing tectal neurons.

### Altered DSCAM expression in the optic tectum impacts visual avoidance behavior

**Fig. 6:**
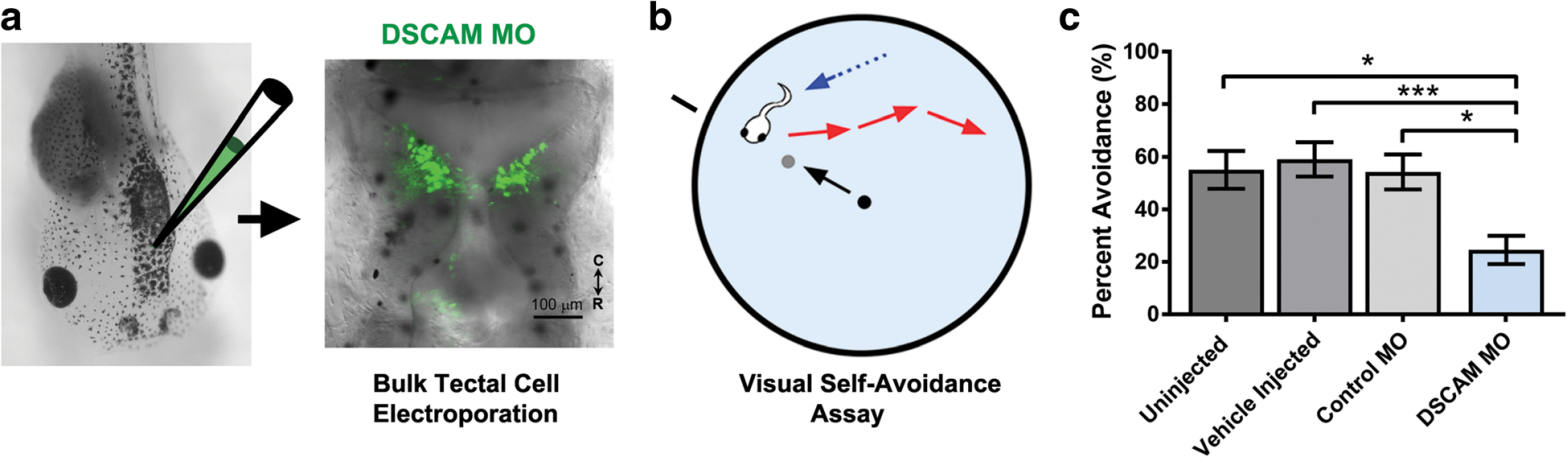
Downregulation of DSCAM expression in the optic tectum affects visually guided behavior. **a** Fluorescein-tagged Control MO or DSCAM MO was bulk electroporated into the caudal midbrain region of stage 43 tadpoles. Fluorescence microscopy imaging was used to confirm bilateral MO transfection into the optic tectum at stage 45. **b** Schematic of the visual avoidance task. The tadpole’s response to the advancing stimuli (black to gray circle) results in the tadpole changing its swimming direction (red arrows). **c** Tadpoles electroporated with DSCAM MO had decreased avoidance responses to the presentation of the stimulus 24 h post-treatment when compared to uninjected controls, vehicle injected controls, and Control MO electroporated tadpoles (Student’s t-test). Error bars indicate mean ± SEM. * *p* ≤ 0.05, ** *p* ≤ 0.005, *** *p* ≤ 0.001. Scale bars: 100 μm (**a**)

To correlate structural changes in tectal neuron morphology mediated by DSCAM missexpression with potential functional changes, we used a modified avoidance task adapted to probe specific visual responses of tadpoles at stage 46. This behavioral assay assessed the effects of downregulating DSCAM levels bilaterally in the optic tectum by targeting the MO transfection specifically to the caudal midbrain (Fig. 6a). Between stages 44 and 47, tadpoles begin to show an avoidance response to moving visual stimuli that is mediated by the maturing retinotectal circuit, which correlates with changes in response properties of tectal neurons [27]. Tadpoles naturally freeze or swim away rapidly when presented with visual stimuli (Fig. 6b). Tadpoles with targeted DSCAM MO electroporation into the optic tectum at stage 45 showed significant deficits in visual responses at stage 46, 24 h after transfection (Fig. 6c). DSCAM MO knockdown significantly decreased the tadpoles’ avoidance behavior when compared to tadpoles transfected with Control MO at the same stage, and with control uninjected or vehicle injected tadpoles (Fig. 6c). No change in swim time was observed for any of the groups tested (not shown). The altered response to visual stimuli therefore indicates that structural cell-autonomous changes in tectal neuron dendritic arbor morphology can impact their connectivity and in turn influence visual information processing in the developing retinotectal system.

**Fig. 7:**
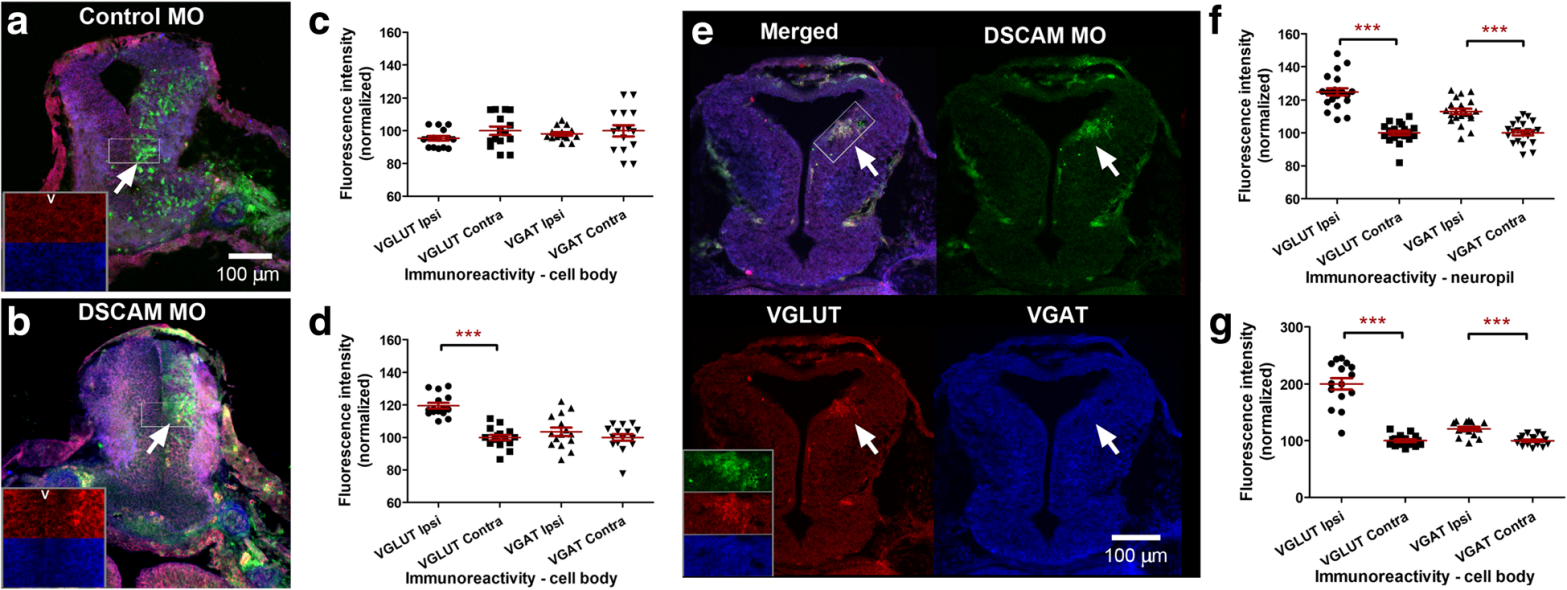
DSCAM downregulation alters excitatory to inhibitory synaptic ratios. **a**, **b** Fluorescein-tagged Control MO or DSCAM MO (*green*) were injected into the light-shaded blastomeres of 4-cell stage embryos; animals were raised to Stage 45. Stage 45 morphant tectal tissues were immunostained with antibodies targeting vesicular glutamate transporter (VGLUT, *red*) and vesicular GABA transporter (VGAT, *blue*). Levels of VGLUT and VGAT immunoreactivity were quantified in midbrain regions with MO (right hemisphere-ipsilateral side; white arrows in (**a** and **b**) and were compared to the contralateral side (left hemisphere) where MO was not present. Fluorescence intensity for VGLUT (red*, top*) and VGAT (blue, *bottom*) immunoreactivities in both hemispheres is also illustrated by the magnified inserts where the ventricle (*v*) demarcates the separation between the ipsilateral and contralateral sides. **c** No significant differences in VGLUT or VGAT fluorescence intensity were detected between the ipsilateral side with control MO and the contralateral side without MO. **d** A significant increase in VGLUT intensity was observed along the cell body layer on the ipsilateral side of the tectum treated with DSCAM MO compared to the contralateral side without MO. **f** VGLUT and VGAT immunoreactivity was also increased in the neuropil ipsilateral to the DSCAM MO label. **e** Targeted bulk electroporation was used to focally transfect fluorescein-tagged Control MO or DSCAM MO into the tectum of stage 42 tadpoles; animals were then raised to stage 45 to compare levels of VGLUT and VGAT via immunohistochemistry. The difference in fluorescence intensity in VGLUT (*red*) and VGAT (*blue*) immunoreactivity in neighboring areas with and without the DSCAM MO fluorescein tag (*green*) is illustrated in the overlap and by separating the individual channels (see also the magnified insert; *bottom left*). **g** Note brain regions electroporated with DSCAM MO exhibited an increase in VGLUT and VGAT intensity relative to the contralateral non-MO side (Student’s t-test). Error bars indicate mean ± SEM. *** *p* ≤ 0.001. Scale bars: 100 μm in (**a**, **b**, **e**)

To further analyze whether structural and functional changes in retinotectal connectivity caused by DSCAM dysregulation correlate with synaptic modifications in the circuit, we determined potential changes in excitatory and inhibitory inputs by immunostaining with antibodies to vesicular glutamate transporter 2 (VGLUT) and vesicular GABA transporter (VGAT) in stage 45 tadpoles. Knockdown of DSCAM expression in embryos at the four-cell stage resulted in a significant increase in VGLUT immunoreactivity in the stage 45 tectal hemisphere where the DSCAM MO fluorescein tag localized (20% relative increase in VGLUT immunofluorescence intensity in hemisphere ipsilateral to MO label versus contralateral side, Control MO, Fig. 7a, c; DSCAM MO, Fig. 7b, d, f). Moreover, targeted DSCAM MO electroporation into the optic tectum at stage 42 resulted in a more significant increase in both VGLUT and in VGAT immunoreactivity in midbrain regions with DSCAM MO label when compared to the contralateral (non-transfected) side of the same tadpoles (Fig. 7e, g). These results indicate that synaptic alterations in excitatory and inhibitory inputs as well as in excitatory to inhibitory balance accompany the changes in tectal neuron dendritic arbor morphology. The observation that altered synaptic connectivity accompanies DSCAM downregulation supports the effects of single-cell MO treatment and indicates that MO-mediated knockdown results in rapid changes in connectivity that may be compensated, at least in part, as neurons and/or the circuits mature*.*

### Retinal ganglion cells exhibit stunted axon branching in response to downregulation of DSCAM levels

**Fig. 8:**
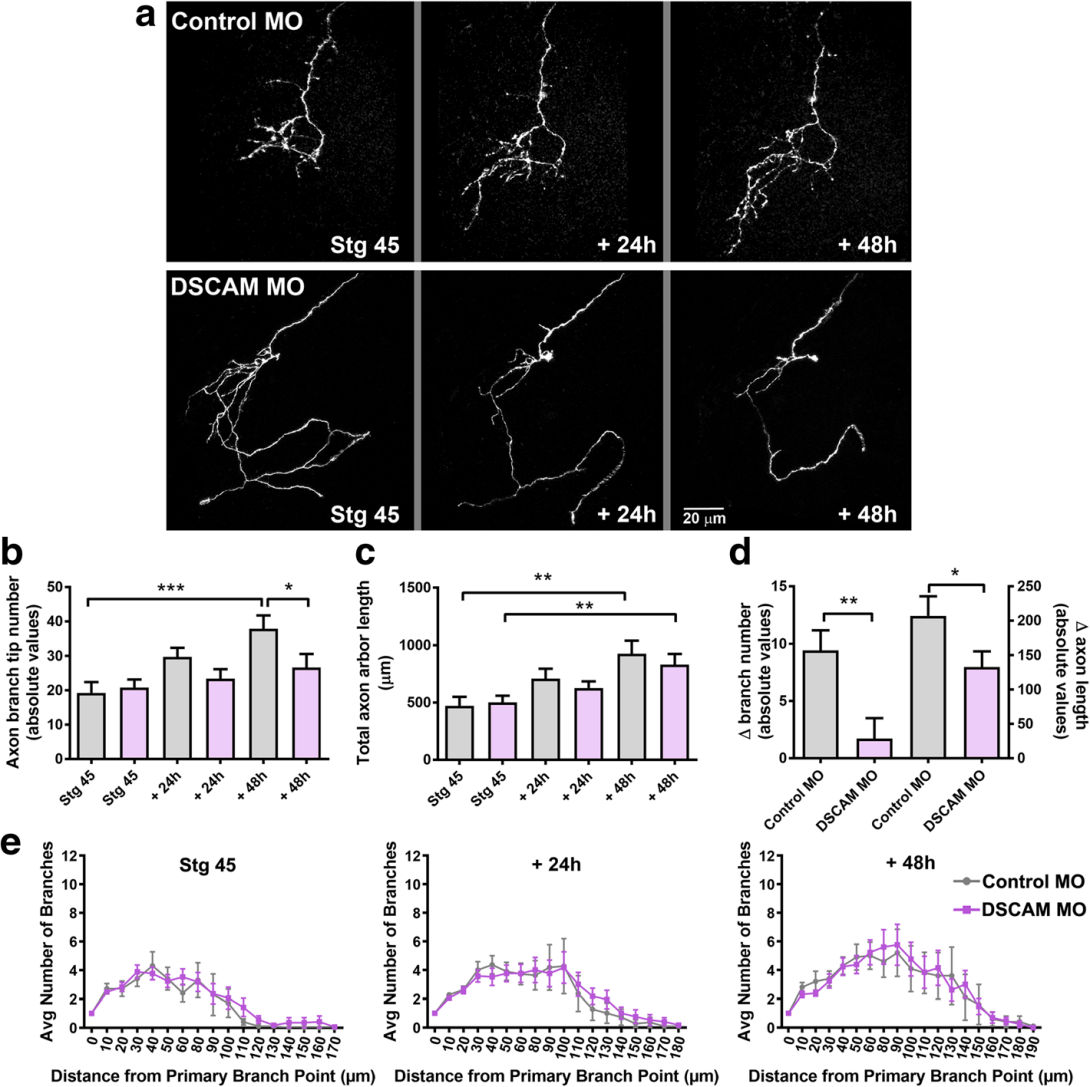
DSCAM downregulation decreases RGC axon arbor growth cell autonomously. **a** Sample axon arbors from RGCs transfected with Control MO or DSCAM MO together with Alexa 488 dextran and imaged at stage 45, and 24 and 48 h after initial imaging. **b**-**d** Quantitative analysis of axon branch number (**b**) and total axon arbor length (**c**) demonstrate that in contrast Control MO transfected RGCs, axons from RGCs with DSCAM knockdown failed to increase their number of branches over time, an effect that significantly decreased axon arbor growth rate (**d**; change in branch number and length). **e** Sholl analysis revealed no significant differences in the branching patterns of RGC axons with DSCAM knockdown each imaging time point when compared to controls. Control MO (*n* = 15) or DSCAM MO (*n* = 18). Comparisons are by Two-way ANOVA and Student’s-t-test. Error bars indicate mean ± SEM. * *p* ≤ 0.05, ** *p* ≤ 0.005, *** *p* ≤ 0.001. Scale bars: 20 μm in (**a**)

DSCAM protein expression localizes to both the retina and optic tectum of developing *Xenopus* tadpoles (Fig. 1) and could therefore also affect synaptic connectivity in the retinotectal system by acting presynaptically. To investigate whether DSCAM independently modulates the targeting and branching of developing presynaptic retinal axon arbors, we examined the effects of DSCAM downregulation in individual RGCs. Co-electroporation of DSCAM MO and Alexa 488 cell-filling dextran in single RGCs of anesthetized tadpoles was used to downregulate DSCAM expression at stage 43, when RGC axons target and begin to branch in the optic tectum. In vivo two-photon confocal microscopy imaging of individual RGC axons 24 h after MO transfection, at stage 45, showed no targeting errors in axons from either DSCAM MO or Control MO transfected RGCs. Axons from RGCs with DSCAM downregulation projected normally to the contralateral tectal neuropil (Fig. 8a). However, both qualitative and quantitative analyses of axons imaged over the course of 3 days showed limited axonal arbor growth in axons of RGCs with DSCAM MO-mediated knockdown. RGC axon arbors with DSCAM knockdown had similar number of terminal branches as control at the first imaging time point but over the course of the 48-h imaging period failed to significantly increase their number of branches (Fig. 8b). While axon arbors of RGCs with DSCAM knockdown continued to lengthen over the 48-h imaging period (Fig. 8a, c), axon arbors exhibited a significant slower growth rate relative to axons of RGCs transfected with Control MO (Fig. 8d, change in branch number and length). Even though axons from RGCs with DSCAM knockdown extended fewer branches over the 48-imaging period, Sholl analysis revealed that the overall distribution of terminal branches of RGC axonal arbors treated with DSCAM MO did not differ from that of RGC axon arbors transfected with Control MO (Fig. 8e). Moreover, axon branches from RGCs transfected with DSCAM MO continued to self-avoid (not shown).

**Fig. 9:**
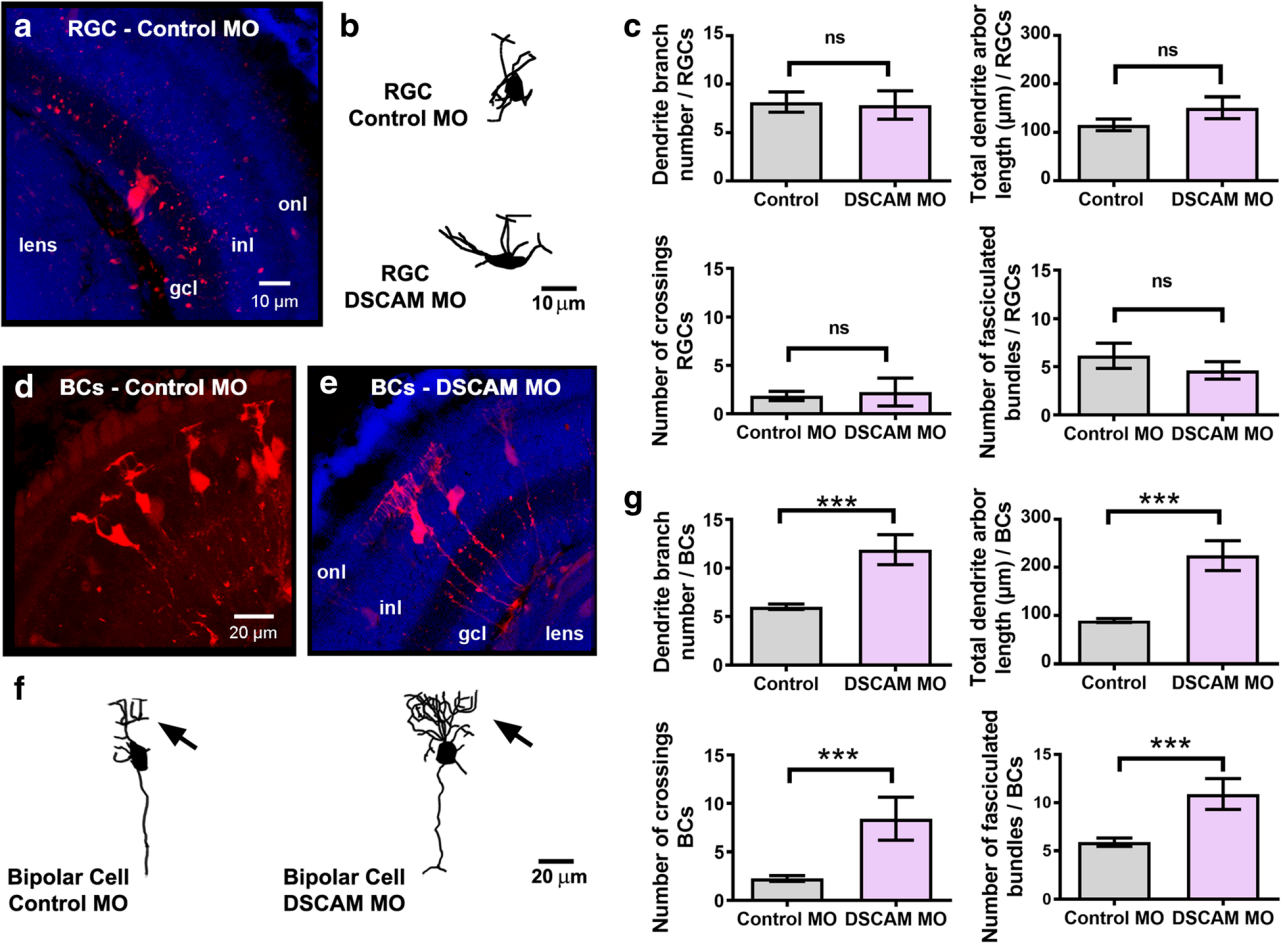
DSCAM downregulation differentially influences RGC and bipolar cell dendrite growth. Dendritic morphologies of fluorescently labeled (**a**-**c**) RGCs and (**d**-**g**) bipolar cells (*BCs*) transfected with Control MO or DSCAM MO are illustrated by the confocal projections of stage 45 retina cryostat sections (***a, d, e***) and sample three-dimensional tracings (***b, f***). Sections in (**a** and **e***)* were counterstained with DAPI to reveal the retinal layers. *Inl,* inner nuclear layer; *gcl,* ganglion cell layer; *onl*, outer nuclear layer. **c** The number of dendritic branches, total dendritic arbor length, number of dendritic crossings, and number of fasciculated dendritic bundles of RGCs treated with DSCAM MO (*n* = 18) were compared to those treated with Control MO (*n* = 13). No significant differences were found across each category. **g** Morphological analysis of neurons traced three-dimensionally reveals a significant increase in the number of dendritic branches and total dendritic arbor length of bipolar cells in response to DSCAM downregulation. BCs treated with DSCAM MO also showed a significant increase in the number of dendritic crossings and fasciculated bundles compared to cells treated with control MO. DSCAM MO (*n* = 28), Control MO (*n* = 42). Error bars indicate mean ± SEM. ***p < 0.001, or *ns* for no significance. Scale bars: 10 μm in (**a**, **b**); 20 μm in (**d**-**f**)

To differentiate effects of DSCAM on dendritic differentiation versus axon arborization on the same population of neurons, namely RGCs, we performed bulk electroporation of either control MO or DSCAM MO in retina of stage 41 tadpoles and analyzed dendritic arbor morphology of sparsely labeled neurons 48 h after treatment. Multiphoton confocal microscopy of fixed stage 45 retinal sections showed that the number of branches and the total length of the dendritic arbors of RGCs with DSCAM MO-mediated knockdown were not significantly different from those of control MO transfected RGCs (Fig. 9a-c). Because electroporation of MO resulted in the sparse transfection and labeling of neurons in the same retinal tissue, we also analyzed bipolar cell dendritic morphologies to confirm the effectiveness of the treatment. This analysis revealed that downregulation of DSCAM in retinal bipolar cells results in significant morphological changes, with neurons possessing a significantly higher number of dendritic branches and longer total dendritic arbor length when compared to control MO transfected bipolar cells (Fig. 9d-g). These observations are consistent with findings on effects of targeted DSCAM knockout on a subpopulation of bipolar cells in the mature mouse retina [4]. To further evaluate potential effects of DSCAM downregulation on dendritic arbor morphology of retinal neurons, we quantified the numbers of dendrite crossings and the number of dendrites that overlap in both RGCs and bipolar cells with DSCAM MO-mediated knockdown. Only bipolar cells showed deficits in dendrite self-avoidance, therefore demonstrating differential effects of DSCAM downregulation that depend on the cell type (Fig. 9c, g). Together, these results indicate that in the *Xenopus* visual system, endogenous DSCAM acts at multiple levels along the visual pathway and independently modulates dendrite and axon arborization of RGCs.

## Discussion

In this study, we examined whether DSCAM directs *Xenopus* retinotectal synaptic connectivity by guiding the structural development of both pre- and post-synaptic arbors. To test cell-autonomous roles of DSCAM, we manipulated DSCAM expression in individual tectal neurons and in RGCs in vivo. Our results show that DSCAM has two dual opposing roles in coordinating the formation of retinotectal connections – DSCAM restricts tectal neuron dendrite arbor growth while facilitates RGC axon arbor development in the midbrain of the *Xenopus* tadpole. We also demonstrate that DSCAM plays a pivotal role in directing dendritic branch pathways of tectal neurons. Finally, we show that the structural development of retinotectal circuits mediated by DSCAM is necessary for proper visual processing. Together, our findings reveal that DSCAM directs pre- and post-synaptic arbor formation during embryonic development and is important for emergent visual function.

The effects of altering DSCAM in tectal neurons suggest that the cell-adhesion molecule serves as a limiting factor that confines dendrite arbor growth during development. Overexpression of either a chimeric protein coding for DSCAM tagged with GFP or DSCAM protein alone in single tectal neurons in otherwise intact tadpoles significantly limited dendrite branching and growth, while MO-mediated knockdown of DSCAM expression resulted in exuberant arbor growth. Effects of DSCAM knockdown were unique and robust, as dendrites branched and took on a tortuous path of growth, significantly increasing arbor size and affecting their connectivity. It is therefore possible that restriction of dendritic arbor size and shape mediated by DSCAM is a result of potential repulsive mechanism [28–31] similar to that facilitated during neuronal tiling [32]. Tiling of arbors are mediated by homotypic repulsive interactions between neighboring cells, limiting arbors to a specific size and space to ensure that arbor territories do not overlap [2]. This tiling arrangement of arbors typically occurs in a two-dimensional laminar space and is a mechanism that modulates neuronal arbor size [1–3, 33]. For example, targeting DSCAM knockout to mature mouse retina has revealed that bipolar cells expand both their dendritic and axonal fields in the absence of DSCAM, suggesting that DSCAM acts as a signaling cue that restricts dendrite and axon outgrowth to preserve tiled arrangement [4]. In *Xenopus,* developing neurons do not tile within the tectal neuropil. Tectal neuron dendritic arbors are, however, quite elaborate in three dimensions (average 50–80 μm in depth) and overlap with neighboring arbor fields extensively. Our results therefore indicate that modulating the size of arbor fields developing three dimensionally within the brain may also be a mechanism by which cell surface proteins such as DSCAM control synaptic connectivity of developing neurons in the visual system.

One unexpected finding was that downregulation of DSCAM expression in tectal neurons or in RGCs did not result in perturbation of self-avoidant branch patterning of dendrites or axons. No clear fasciculation of sister dendrite branches among arbors were observed in these neurons, although such phenotype was observed for dendrites of retinal bipolar cells with DSCAM knockdown. In the *Drosophila* peripheral nervous system, isoform-specific homophilic interactions of DSCAM trigger sister dendrite repulsion. This cellular organization occurs in a stereotypic manner and prevents the overlapping of neuronal dendrites from the same neuron while allowing dendrites from different cells to overlap in the neuropil [10, 23]. Dendritic self-avoidance is made possible due to the thousands of isoforms of DSCAM that *Drosophila* can express and that is facilitated through mRNA alternative splicing [5]. *Xenopus,* as other vertebrate species, is known to express only two isoforms of DSCAM – DSCAM and DSCAML1 – that are coded by two distinct genes. We specifically altered expression of *Dscam*, the gene implicated in Down syndrome, to study its central function during vertebrate visual system development. Our real time imaging experiments demonstrate that while in *Xenopus* DSCAM does not mediate self-avoidant organization of dendritic and axonal arbors at retinotectal synapses, it differentially shapes both presynaptic RGC and postsynaptic tectal neuron arbors. In our studies, no clear fasciculation of sister dendrite branches among arbors were observed in tectal neurons with DSCAM knockdown. However, altered dendrite self-avoidance was observed in developing *Xenopus* retinal bipolar cells but not in RGCs. Analysis of mutant mice has shown that both *DSCAM* and *DSCAML1* are involved in dendrite self-avoidance in the retina, with DSCAM influencing bipolar cells, amacrine cells and RGCs [4, 11, 34]. In *Xenopus*, dendritic arbors of bipolar cells normally self-avoid and arborize compactly in three-dimensional space (Fig. 9d, average 8–10 μm in depth). DSCAM knockdown affected these two processes, increasing the number of branches that overlap as well as branch number. Therefore, the effects of targeted DSCAM downregulation in developing *Xenopus* bipolar cells are consistent with findings of effects of targeted DSCAM downregulation in mature mouse bipolar cells [4].

The observation that knockdown of DSCAM expression in *Xenopus* RGCs decreased axon terminal branching but did not alter dendrite number or induced dendrites to overlap may be due to cell-type and species-specificity of the effects, or alternatively due to the developmental stage of their dendritic arbors or the timing of DSCAM knockdown. Among its multiple functions in the retina of distinct vertebrate species, DSCAM has been implicated in modulating dendrite self-avoidance and in guiding dendrites to stratify in specific synaptic laminae. A role for DSCAM in synaptic lamination of RGC dendrites within the inner plexiform layer was first demonstrated in the developing chick retina through manipulations of *Dscam* expression, while in *Dscam* and *DscamL1* knockout mice laminar specificity seemed to be preserved [11, 12]. More recent cell-type-specific analyses of DSCAM function have revealed some similarities in DSCAM’s role in synaptic lamination of RGC dendrites among vertebrate species, as defects in lamination can be induced by non-autonomous changes in DSCAM expression in mice [35]. The influence of DSCAM in the spatial organization and fasciculation of dendrites of the same cell type has also been demonstrated for retinal neurons in mice through cell-type-specific loss and gain of DSCAM function [11, 36]. In mouse RGCs, the role of DSCAM in self-avoidance appears to be restricted to neurons of the same type, guiding them as they extend processes and encounter the distal processes of neighboring homotypic cells [35, 36]. In stage 45 *Xenopus* tadpoles, the dendritic arbors of RGCs are still quite immature, extending only a few short dendrites towards a developing inner plexiform layer, thus effects of DSCAM dysregulation on dendrite fasciculation, branching or shape may not transpire within the short period of MO-mediated downregulation of DSCAM expression. In contrast to their dendrites being unaffected, downregulation of DSCAM expression in RGCs significantly impacted the arborization of their axons at the target at the same developmental stage.

Several studies using mouse models have shown that DSCAM is implicated in several aspects of optic pathway development. DSCAM has been implicated in the growth of RGC axons from the chiasm to the dorsal thalamus, with axon arrival at the target site being delayed in DSCAM knockout mice [6]. Moreover, analysis of a mouse model of Down syndrome shows that DSCAM organizes the segregation of ipsilateral and contralateral retinal axons in the dorsal lateral geniculate nucleus [37]. These findings suggest that DSCAM promotes RGC axon growth and controls the timing of when RGC axons reach their visual brain target sites. Our studies demonstrate a novel, cell-autonomous role for DSCAM during RGC axon growth and arborization at their target that is independent of its potential effects on their dendritic arbor. In *Xenopus,* RGC axons branched and grew at a slower rate within their target neuropil in response to DSCAM downregulation. These effects were opposite to those of DSCAM downregulation in tectal neurons, where dendritic arbors overgrow, and neurons extend multiple axon terminals. While no obvious targeting errors were observed in individual RGC axons with DSCAM knockdown, errors in axons being able to exit the eye were observed when overexpressing DSCAM in developing RGCs of young tadpoles (data not shown), consistent with observations of misdirected RGC axons within the retina of adult DSCAM mutant mice [38]. An instructive role for DSCAM on presynaptic arbor growth that is independent of its effects on dendrites has been demonstrated for *Drosophila* sensory neurons, where *Dscam* expression levels and homophilic interactions correlate with patterned presynaptic arbor branching and size [39, 40]. Thus, our findings in developing *Xenopus* embryos together with studies that analyzed more mature visual circuits in mice support the notion that DSCAM plays a multifaceted role in modulating the growth and timing at which RGC axons reach and arborize in brain targets for precise visual connections to form. These studies also demonstrate that the function of DSCAM on RGC axon terminals is separable from its dendritic functions, at least during early stages of dendritic and axon arbor development.

Multiple complementary molecular and signaling mechanisms are involved in dendrite differentiation and arborization that may vary depending on cell type [41–44]. DSCAM has been implicated as a netrin receptor that collaborates with DCC and traffics commissural axons across the ventral portion of the spinal cord [45]. Moreover, studies have shown that DSCAM-netrin signaling is involved in mechanisms driving axon attraction towards their target site [45–48]. While DSCAM and DCC collaborate as co-receptors at the axon terminal in the spinal cord [45], roles for DSCAM during dendritic arbor development appear to be independent from netrin signaling, as shown by their differential effects on the targeting of dendrites in the *Drosophila* CNS [49, 50]. In the *Xenopus* visual system, netrin signaling is an important factor that modulates several aspects of retinotectal development [19, 51–53]. Previous work from our laboratory has shown that netrin influences not only pathfinding, branching, and synaptic differentiation of mature RGC axons at their target [51, 52], but also that acute alterations in netrin levels can rapidly induce postsynaptic remodeling of tectal neuron dendritic arbors, with tectal neuron dendrites remaining simple over time and redirecting their directionality of growth when netrin levels are increased or receptor signaling is altered [19]. Our current studies support the idea that DSCAM acts independent of netrin signaling during tectal neuron differentiation rather than as a canonical receptor for netrin-1. Cell-autonomous downregulation of DSCAM expression resulted in neurons with exuberant dendritic arbor growth, an effect that significantly differs from altered DCC-mediated netrin signaling. Downregulation of DCC levels in the optic tectum with function blocking antibodies to DCC [19], and knockdown of DCC expression in single tectal neurons through DCC MO transfection (A.N. Nagel and SCC, unpublished data) both result in altered directionality of dendrite arbor growth, an effect that differs from the effects of either DSCAM downregulation and overexpression. Thus, our results demonstrate that in the retinotectal system DSCAM is required for proper arbor development of pre- and postsynaptic neurons that are themselves modulated by netrin-dependent signaling, but that DSCAM acts independently of DCC signaling. Whether in RGC axons DSCAM participates, at least in part, in netrin-mediated DCC signaling remains a possibility since downregulation of DSCAM expression in RGCs interfered with RGC axon branching, similarly to effects of altering DCC signaling at the optic tectal target [51, 52].

It is known that the size and shape of a dendritic arbor is important in modulating the degree of connectivity and the neuron’s encoding capabilities [54]. If DSCAM modulates the shape and size of individual central neuron dendritic arbors, these structural changes in arbor size have the potential to alter the neurons’ ability to normally encode visual information. Our results indicate that DSCAM can impact responses to visual stimulus not only by modulating local retinal circuitry but also by shaping neuronal connectivity to optimally control the integration of visual function centrally. Our visual behavioral assay showed that downregulating DSCAM exclusively in a population of neurons in the optic tectum resulted in a decrease of a tadpole’s avoidance responses to a visual stimulus. The behavioral test allowed us to observe effects of cell-specific alterations in DSCAM expression on normal visual function during a precise period of development. This serves as an advantage over traditional knockout studies where effects are observed in more mature animals well after circuits are formed and key developmental processes have already occurred. Our observations that tectal knockdown of DSCAM elicits deficits in the tadpole’s ability to process visual information thus indicate that structural cell-autonomous changes mediated by DSCAM influence central neuron functional connectivity in the developing vertebrate visual system.

Dysregulated DSCAM expression in the optic tectum resulted in changes in visual behavior of tadpoles that may not only be explained by the structural changes in tectal neurons but also by synaptic changes within the circuit. Proper synaptic transmission across circuits depends, at least in part, on the morphology of dendritic arbors [55, 56]. Studies investigating the physiology of circuits have demonstrated that structural dendritic arbor changes affect neuronal excitability [57–59]. In addition to assessing visually guided behavior in tadpoles with DSCAM knockdown, we indirectly correlated structural dendritic changes to synaptic changes by examining VGLUT/VGAT expression as a proxy of synaptic changes in tectal neurons. Our results demonstrate that synaptic changes in excitatory markers (VGLUT) in the optic tectum are more significant than the changes in inhibitory markers (VGAT) and accompany the exuberant changes in dendritic growth of tectal neurons when DSCAM expression is downregulated. Thus, reduction in DSCAM expression can alter synaptic balance and neuronal excitability of tectal neurons – either by directly modulating glutamate receptors or VGLUT/ VGAT transmission at the synapse, or indirectly by changing the structural pattern of dendritic arbors which can consequently affect visual responses corresponding to tectum-dependent visual behavior. It is possible that DSCAM signaling may also be acting on multiple mechanisms simultaneously, coordinating transmission at the synapses and patterning the structure of dendritic arbors at the cellular level, like in *Aplysia*, where DSCAM signaling can directly modulate neuronal activity at the synapse by altering glutamate receptor expression during learning-related synapse formation [60].

An emerging concept is that molecules that participate in neuronal wiring and that are aberrantly expressed in Down syndrome may differentially impact multiple cell-types, may affect each cell type at different times in development, and may continue to affect neuronal function even in the adult CNS [4, 38]. A leading cause of abnormal cognitive and sensory disabilities in individuals with Down syndrome has been attributed to aberrant changes in neuronal wiring during human embryonic development. It is therefore possible that DSCAM overexpression may contribute to changes in early neuronal wiring at multiple levels along the visual pathway that significantly affect cognitive and sensory functions later in life [45]. Indeed, infants with Down syndrome show deficits in spatial visual acuity and contrast sensitivity that have been linked to abnormal wiring of visual circuitry [45, 61]. Our studies in *Xenopus* for the first time implicate DSCAM in the control of both pre- and postsynaptic structural and functional connectivity in the developing visual system, where it differentially guides postsynaptic dendrite growth of neurons in the central visual targets while it also facilitates presynaptic arborization of RGC axons acting cell-autonomously. Determining the cell-autonomous contribution of DSCAM to early aspects of neural circuit formation at a single population level in accessible vertebrate animal models can help better understand the pathophysiology of complex neurodevelopmental disorders that affect neural circuit formation and function.

## Conclusion

*Xenopus laevis* was used as a model to examine developmental effects of DSCAM in vivo and to provide a unique temporal and spatial understanding of how visual circuits are dynamically shaped. In the *Xenopus* visual system, endogenous DSCAM acts at multiple levels along the visual pathway and independently modulates dendrite and axon arborization, where cell-autonomous roles vary depending on the cell type. Our observations implicate DSCAM in the control of both pre- and postsynaptic neuronal cytoarchitecture and functional connectivity in the retinotectal circuit, whereby it primarily acts as a neuronal brake to limit and guide tectal neuron dendrite growth. RGC axons at the target are differentially influenced by DSCAM, where DSCAM expression levels positively impact presynaptic arbor size. The cellular mechanisms mediated by DSCAM in shaping tectal neuron connectivity also play a key role in central visual processing. Thus, the wiring of functional neural circuits during embryonic development requires coordinated organization between developing axon and dendritic arbors, a process that is dependent on molecules that have been implicated in Down syndrome and autism, such as DSCAM.

## Abbreviations

ANOVA: Analysis of variance
BC: Bipolar cell
DSCAM: Down syndrome cell-adhesion molecule
MO: Morpholino antisense oligonucleotide
RGC: Retinal ganglion cell
VGAT: Vesicular GABA transporter
VGLUT: Vesicular glutamate transporter
